# Tracheobronchial Stenoses in Granulomatosis With Polyangiitis (Wegener's): A Report on 26 Cases

**DOI:** 10.1097/MD.0000000000001088

**Published:** 2015-08-14

**Authors:** Charlotte Girard, Pierre Charles, Benjamin Terrier, Guillaume Bussonne, Pascal Cohen, Christian Pagnoux, Vincent Cottin, Jean-François Cordier, Loïc Guillevin

**Affiliations:** From the Department of Internal Medicine (CG, PaC, BT, GB, LG), National Referral Center for Rare Autoimmune and Systemic Diseases, Cochin Hospital; INSERM U1060 (CG, PaC, BT, GB, LG), Hôpital Cochin, Assistance Publique-Hôpitaux de Paris, University of Paris 5-René-Descartes, Paris; Department of Internal Medicine (CG), Department of Rheumatology, Mount Sinaï Hospital, Toronto, Ontario, Canada (CP), Edouard-Herriot University Hospital, Lyon; National Referral Center for Rare Pulmonary Diseases (VC, J-FC), Louis-Pradel Hospital, Lyon, France; and Department of Internal Medicine (PiC), Institut Mutualiste Montsouris, Paris.

## Abstract

Tracheobronchial stenoses (TBSs) are potentially severe manifestations of granulomatosis with polyangiitis (Wegener's) (GPA) that usually respond poorly to corticosteroids and immunosuppressive agents. We describe 26 GPA patients with ≥1 tracheal (mainly subglottic, SGS) and/or bronchial stenosis(ses) (BS(s)).

Sixteen patients had solitary SGS and 10 had ≥1 BS(s). The male/female sex ratio was 9:17, and the median age at GPA diagnosis was 32 years (3:13 and 28 years, respectively, for SGS patients). Antineutrophil cytoplasm antibodies were proteinase 3-positive in 65.5% of the patients (50% of those with SGS).

Despite conventional GPA therapy, 62% patients experienced ≥1 stenosis relapse(s) (81% of SGS patients, for a total of 1–8 relapses per patient). None of the several systemic or endoscopic treatments prevented future relapses. Cyclophosphamide induction therapy was effective in 4/6 patients with BS(s) and in 1 patient with SGS among the 7 treated. After many relapses, rituximab achieved remission in 3/4 SGS patients. Endoscopic treatments (dilation, laser, corticosteroid injection, etc.) had only transient efficacy. Other GPA manifestations relapsed independently of TBSs. One SGS patient died of acute respiratory distress syndrome.

Our findings confirmed that TBSs are severe GPA manifestations that evolve independently of other organ involvements and do not respond to conventional systemic regimens. As previously described, our population was younger and comprised more females than usual GPA patients, especially those with SGS.

The small number of patients and the wide variety of local and systemic treatments prevent us from drawing definitive conclusions about the contribution of each procedure. However, cyclophosphamide seemed to effectively treat BSs, but not SGS, and rituximab may be of interest for SGS management.

## INTRODUCTION

Granulomatosis with polyangiitis (Wegener's) (GPA) is a small-vessel necrotizing granulomatous vasculitis. Ear, nose and throat (ENT), lung, and kidney are the most frequently involved organs (90%, 90% and 80% patients, respectively).^1^ GPA can be life-threatening, but dramatic improvement has been obtained since corticosteroids and immunosuppressive or immunomodulating agents have been prescribed. Initial induction therapy now achieves GPA remission in ∼90% of the patients.

Tracheobronchial involvement, a less common GPA manifestation, comprises stenoses of the tracheobronchial tree, which can lead to upper airway obstruction and potentially severe functional but also life-threatening consequences.^2^ Subglottic stenosis (SGS) is the most frequent tracheobronchial stenosis (TBS) type that could be related to anatomic specificities and pathophysiological mechanisms.^3^ SGS treatment is not codified and is often less effective than that of other systemic manifestations. SGS occurrence and outcome can be independent of systemic GPA manifestations and their evolution.^2,4^ Little is known about bronchial stenoses (BSs) in terms of therapeutic response or evolution.^5^ Endoscopic treatments are frequently needed to improve airway flow.^4,6^

The objectives of this study were to describe patients managed for TBS(s) in 2 French medicine departments, and to compare the features and treatment responses of SGSs versus BSs.

## PATIENTS AND METHODS

We retrospectively identified GPA patients with ≥1 TBS(s) that had been followed and treated in 2 university hospitals (Paris and Lyon, France) during the past decade. GPA diagnosis was based on clinical, radiological, biological, and, sometimes, histological findings. Patients satisfied American College of Rheumatology classification criteria^7^ and Chapel Hill Nomenclature definitions.^8^ This study was conducted in compliance with good clinical practices and the Declaration of Helsinki principles. In accordance with French law, formal approval from an ethics committee is not required for this type of study.

Tracheal stenoses comprised SGSs (located at the level of cricoid cartilage and/or upper tracheal rings, 1.5–2 cm below the true vocal cords and above the trachea) or distal tracheal stenoses (dTSs).^3,9^ BSs affected the main or lobar bronchi (≥1). Tracheal and bronchial stenoses could coexist in the same patient. The diagnosis of stenosis was retained based on a persistent localized narrowing of the tracheal and/or bronchial lumen(s) during exploratory endoscopy or imaging. Each GPA patient identified in the databases of the 2 participating departments as having at least 1 of these stenoses was included in the study.

We paid particular attention to compare patients with isolated SGS versus those with BS(s) with or without SGS or dTS.

Patients’ clinical, biological, histological, and radiological records were reviewed. Data concerning TBSs and systemic manifestations over time and responses to treatments were collected. In particular, each stenosis relapse was identified, and patient's characteristics at that time (systemic disease activity, antineutrophil cytoplasm antibody (ANCA) status, current medications, etc.), systemic and endoscopic treatments, and outcomes were recorded.

Stenosis remission was defined as the disappearance or regression of stenosis-related clinical symptoms (hoarseness, dyspnea, cough, etc.), associated with enlargement of the tracheal or bronchial lumen at the end of the endoscopic procedure or during exploratory endoscopy performed weeks after starting medical treatment. Relapse corresponded to each recurrence after remission of persistent, progressive, specific symptoms, concomitant with stenosis worsening compared to the last explorations.

Disease activity was assessed with the Birmingham Vasculitis Activity Score (BVAS),^10^ whereas sequelae were evaluated with the Vasculitis Damage Index (VDI).^11^

### Statistical Analyses

Descriptive statistics included numbers (percentages) for categorical variables and mean or median [range] for continuous variables. Categorical variables were compared with the nonparametric Fisher exact test. Continuous variables were compared using the nonparametric Mann–Whitney test. For all analyses, the α risk was set at 5%. All analyses were computed with the R v2.4.2 software package (http://www.R-project.org).

## RESULTS

### Patients

**FIGURE 1:**
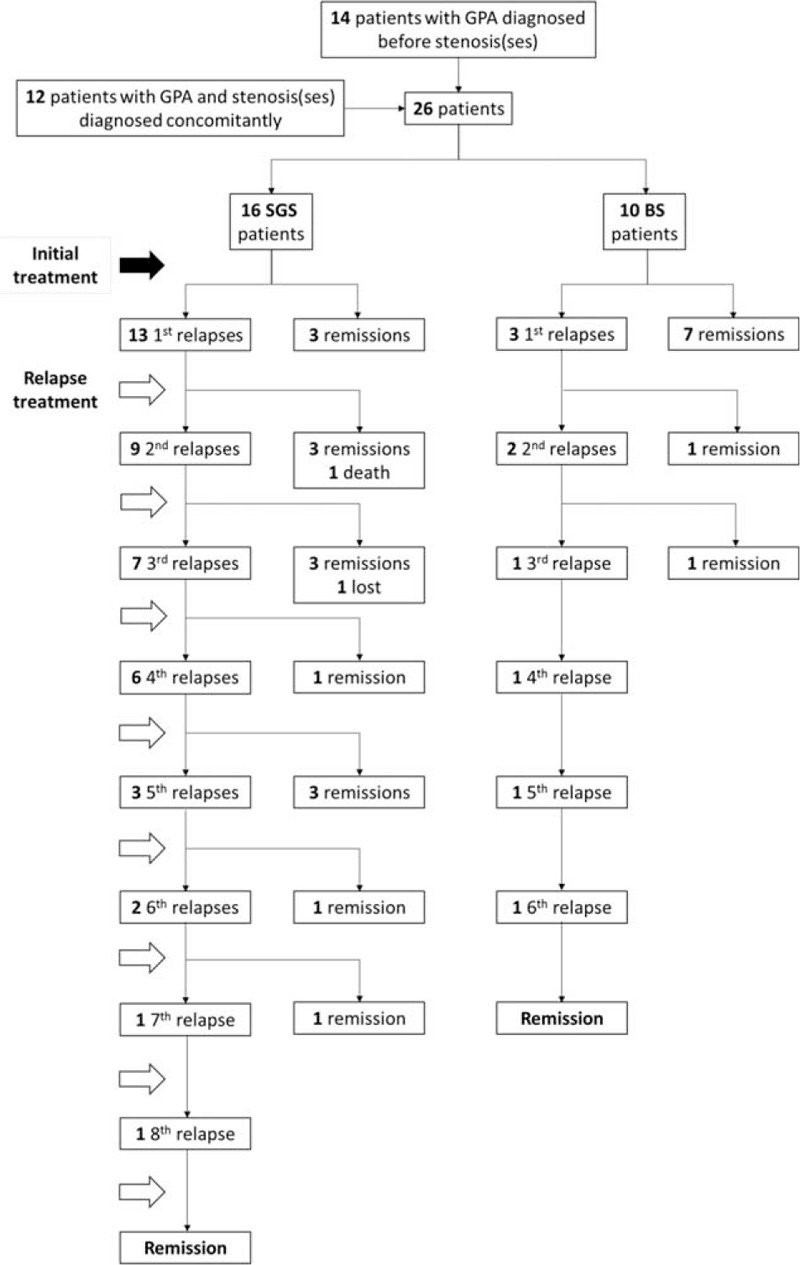
Flow chart from diagnosis of GPA to remission of patients with a SGS or ≥1 BS. The solid black arrow represents the onset of stenosis treatment (and sometimes GPA) at diagnosis. The black-outline arrows are the treatments of each relapse. BS = bronchial stenosis, GPA = granulomatosis with polyangiitis (Wegener's), lost = lost sight, SGS = subglottic stenosis.

**FIGURE 2:**
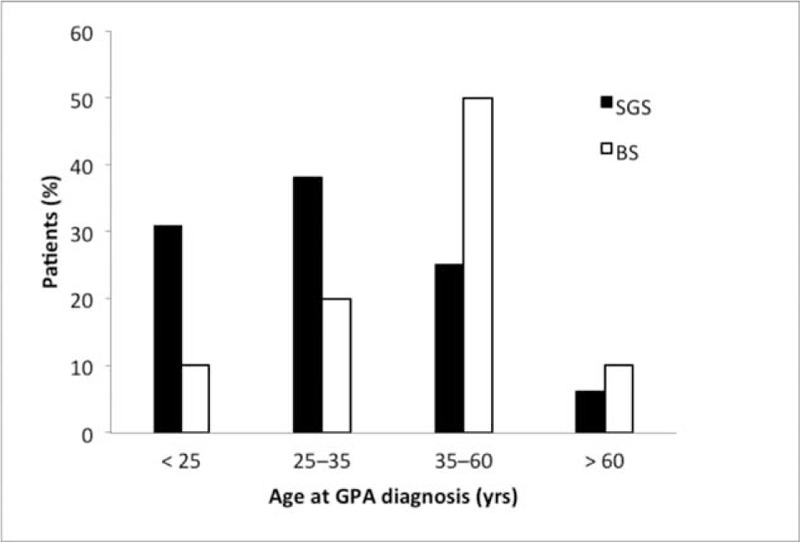
Age at diagnosis of GPA, according to stenosis type. BS = patients with ≥1 bronchial stenosis(ses), GPA = granulomatosis with polyangiitis (Wegener's), SGS = patients with single subglottic stenosis.

Twenty-six patients were included (Figure 1). Their main characteristics are shown in Tables 1 and 2. They were 9 men and 17 women (male/female sex ratio, 0.53). Their median age at GPA onset was 32 (range 6–79) years. Figure 2 shows the patients’ distribution according to age and TBS type.

**TABLE 1:**
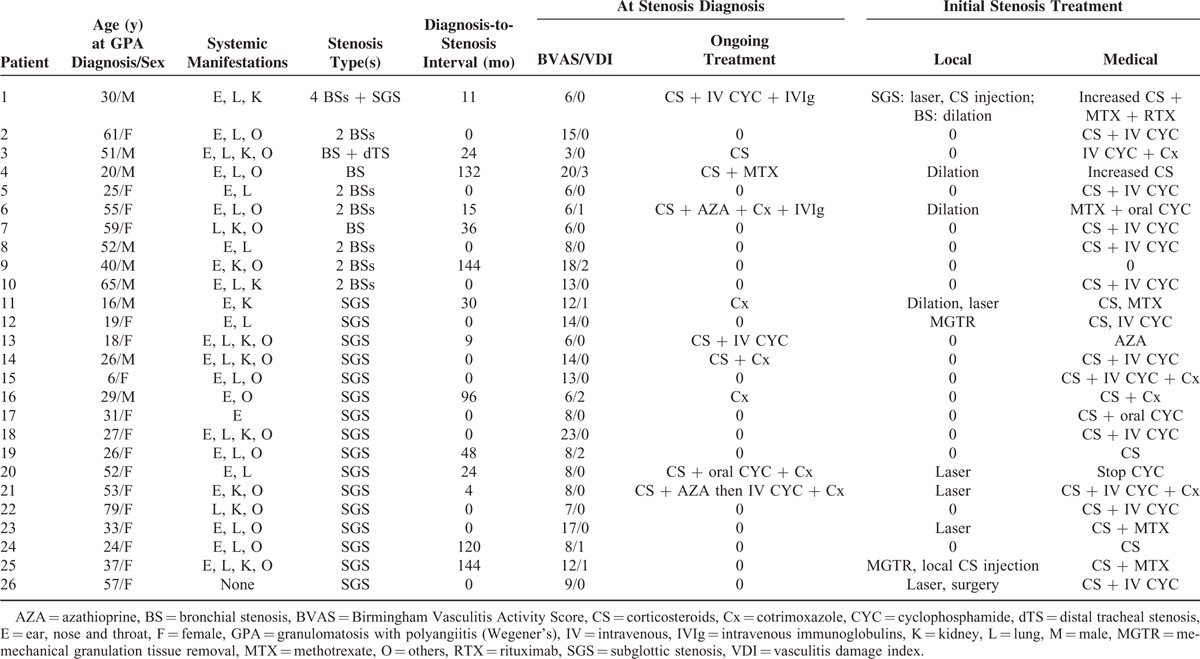
Stenosis Diagnosis and Initial Treatment(s) for Each Patient

**TABLE 2:**
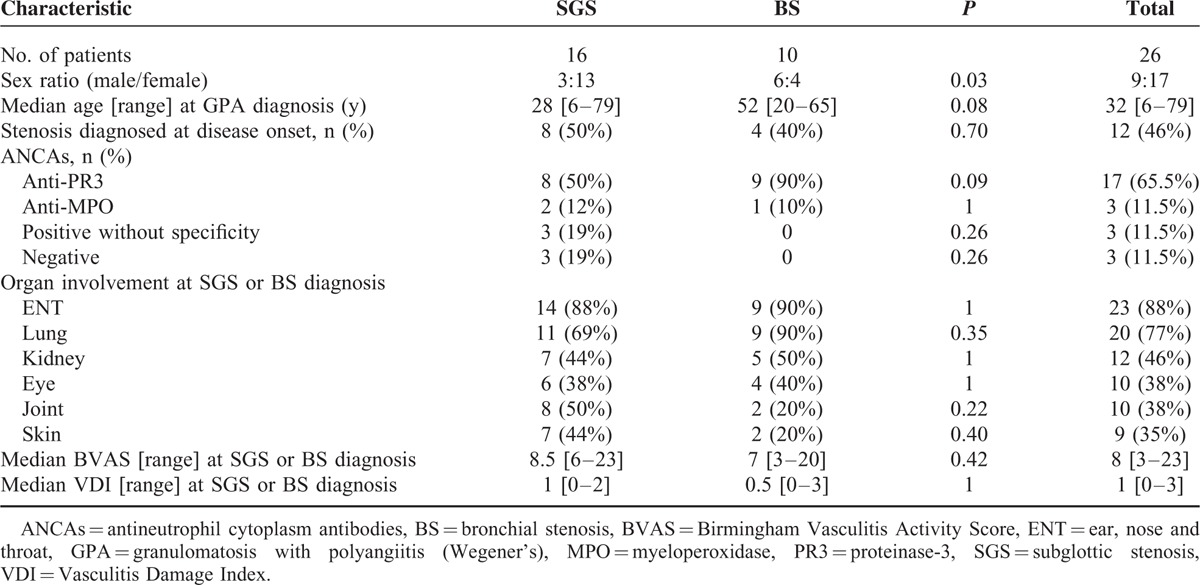
Characteristics of Patients With SGS Versus Those With at Least 1 BS

### Systemic Manifestations

Among the 26 patients, 25 (96%) had systemic GPA manifestations (Tables 1 and 2): 23/26 (88%) patients had ENT involvement (crusty rhinitis, chronic sinusitis, or otitis in most cases), 20 (77%) had associated pulmonary parenchymal manifestations, and 12 (46%) had renal disease. General symptoms (fever, weight loss, asthenia, etc.) were present in 19/26 (73%) patients.

### TBSs

Sixteen patients had isolated SGS (without BSs) and 10 had ≥1 BS(s). Multiple BSs were found in 7/10 patients (Table 1). All but 1 of the 10 BS patients had involvement of the left bronchi and 4/10 of the right bronchi. Left upper and lower lobar bronchi were the most often affected (5 patients each). No patient had isolated dTS, but BS patients 3 and 1, respectively, also had dTS and SGS.

Stenotic lesions were present at GPA onset (12 [46%] patients) or appeared subsequently (14 [54%] patients), after a median of 33 (5–113) months (39 [6–112] months for SGS, 30 [0–120] months for the others) (Table 1).

Patient 1's first identified BS was diagnosed 3 months after his SGS. Patient 3's BS and dTS were diagnosed concomitantly after GPA.

Clinical SGS manifestations were dyspnea on exertion (8/16, 50%), stridor (3/16, 19%), or persistent cough or hoarseness (1 patient each, 6%). Initial BS symptoms were hemoptysis (2/10, 20%), dyspnea or cough (1 patient each, 10%). BSs were asymptomatic in 6 (60%) of the 10 patients, and SGS in 2 (13%) of the 16 patients. In those 8 asymptomatic patients, TBSs were diagnosed fortuitously during the initial GPA work-up or during its monitoring by computed tomography (CT) scan in patient 1, or bronchoscopy in the 7 other patients, which had been motivated by CT identification of pulmonary nodules in 6 patients, or exploration of an intraalveolar hemorrhage in the seventh patient.

### Relapses

Sixteen patients (62%) experienced ≥1 TBS relapse(s) (Figure 1, Table 3), for a total of 51 TBS relapses (42 SGS and 9 BS relapses). Eight (89%) of the 9 BS and 22/42 (52%) SGS relapses occurred while GPA was in remission. ENT disease was the only GPA manifestation during all the remaining TBS relapses, except 4 SGS relapses that were concomitant with eye (scleritis) or joint involvement.

**TABLE 3:**
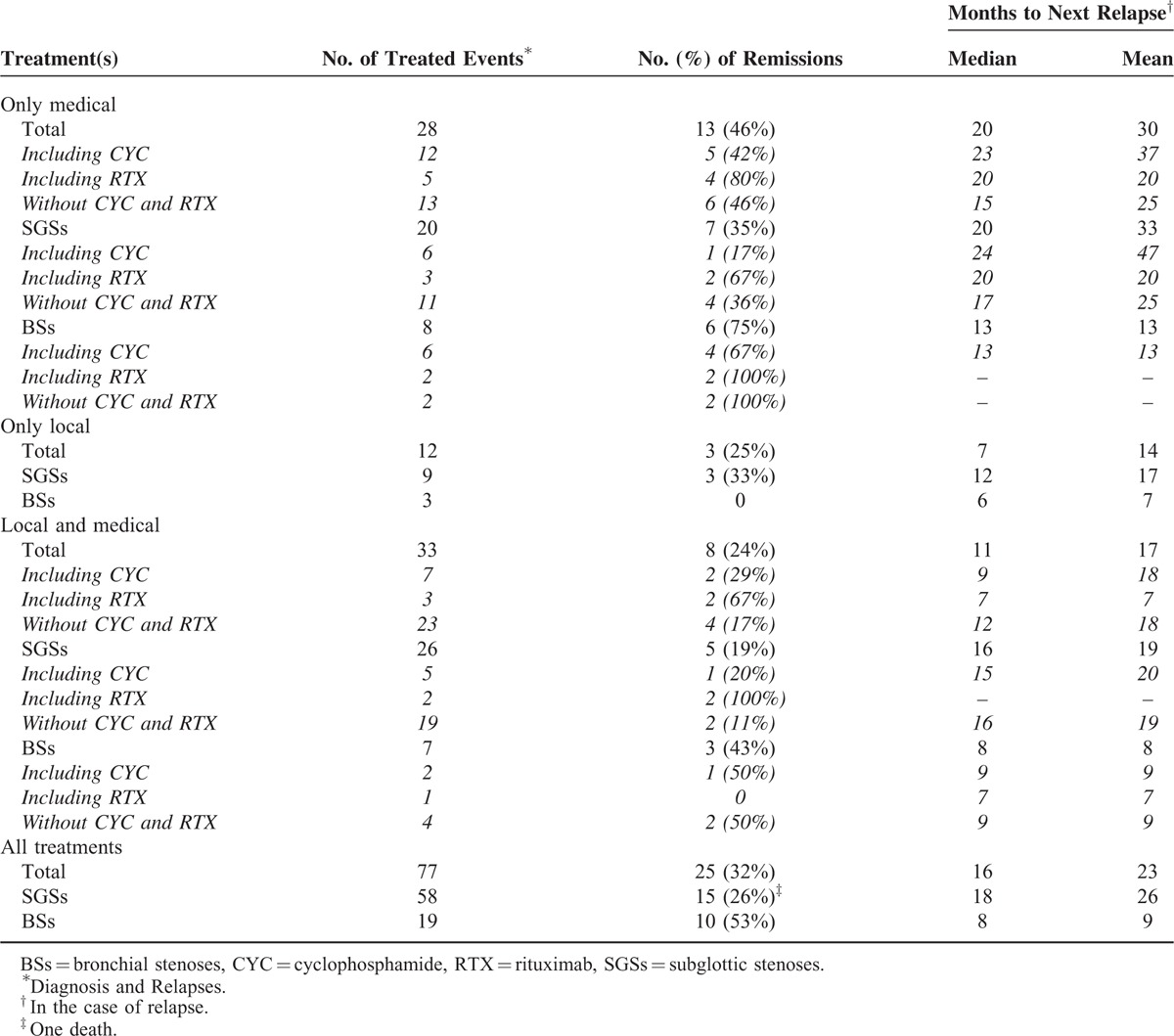
Follow-Up and Relapse Features

Ten (63%; 1 with BSs and 9 with SGS) of the 16 TBS-relapsing patients remained in systemic GPA remission from TBS diagnosis until the end of follow-up. Six (23%) of the 26 patients suffered systemic disease relapses (mostly ENT) without any TBS recurrence.

Patient 1, the only who had BSs and SGS, experienced 6 BS relapses (5 of them in the left main bronchus) and 6 SGS relapses, with 3 of the latter occurring without any concomitant BS relapse.

Among SGS patients, patient 17 was lost sight after 2 relapses, and patient 22 died after her first relapse, of acute respiratory distress caused by obstructive SGS.

### Complementary Investigations

#### ANCA

Twenty-three (88%) patients were ANCA-positive at the time of GPA diagnosis (14 with cytoplasmic ANCA (c-ANCA)- and 4 with perinuclear-labeling patterns, 5 with undetermined specificities). Anti-proteinase-3 (PR3) ANCAs were detected in 17 (65.5%) patients, anti-myeloperoxidase (MPO) in 3 (11.5%), and no specificity in 3 (Table 2).

ANCAs were sought during 21/51 TBS flares (3/9 BS and 18/42 SGS relapses). They were never detected for patients with BS(s) and were negative during 8/18 (44%) SGS relapses. Among the 10 positive ANCA tests during SGS relapses, half had low titers.

#### Histology

**FIGURE 3:**
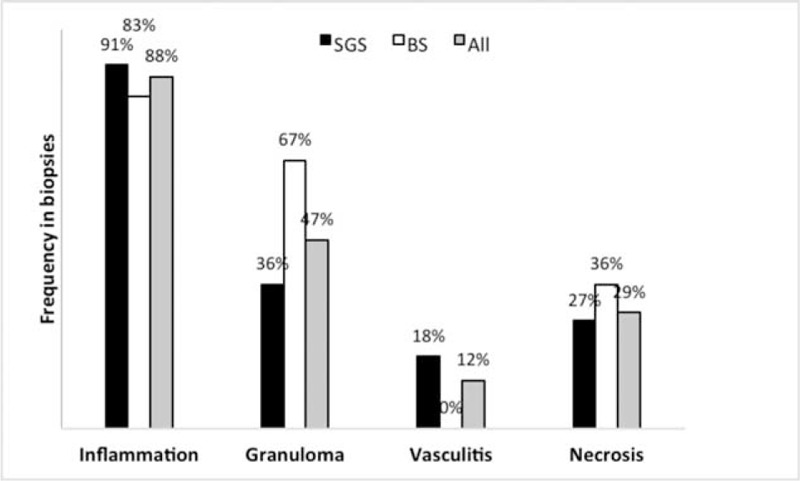
Distributions of histological findings in the biopsies from 11/16 patients with SGS and 6/10 patients with ≥1 BS(s). The percentages indicate the frequency of each histological finding among SGSs, BSs, and all tracheobronchial stenoses (All). BSs = bronchial stenoses, SGS = subglottic stenosis.

Seventeen patients underwent ≥1 tracheal or bronchial biopsies at the time of TBS diagnosis (6/10 BSs, 7/16 SGSs), or at relapse (4/16 SGS patients). Distributions of histological findings are reported in Figure 3. Among the 26 biopsies performed, all but 2 were pathological (92%). Those latter 2 biopsies were obtained, a few months apart, from the same patient. Histological findings were never GPA-specific and never led to the diagnosis.

#### Radiological and Functional Tests used for TBS Management

As of TBS diagnosis, 14/16 SGS patients underwent ≥1 nasofibroscopic examination(s) and 5/10 patients with BS(s) underwent bronchoscopy at least once during their follow-up. CT scans were obtained for 11/16 SGS patients and 6/10 with BSs. Pulmonary function was assessed with flow-volume loop in 16 patients (11 with SGS and 5 with BS[s]). It showed flattening of both limbs of the loop, reflecting fixed upper airway obstruction. Patient 23 with SGS and patient 6 with BSs underwent positron-emission tomography (PET): no uptake was seen at the levels of the stenoses. Magnetic resonance imaging (MRI) was never done.

Aside from being used for monitoring, endoscopy was the investigation of choice for diagnosis (9/10 BSs and 12/16 SGSs). For patient 1 with multiple BSs, and SGS patients 11 and 13, this is CT scan that provided TBS diagnoses.

### Treatments

#### Initial Treatment

Table 1 shows the local and systemic treatments initiated when TBS(s) were diagnosed. Among the 14 patients whose TBS(s) had been diagnosed during the course of GPA, 10 (71%) were already on systemic therapy, comprising oral corticosteroids for all but patients 11 and 16, combined with cytotoxic drug(s). Patients 11 and 16 received cotrimoxazole alone (800 mg × 2 daily). Concerning the 12 patients whose TBS(s) were diagnosed at the same time as the first GPA flare, corticosteroids, and intravenous (IV) cyclophosphamide were prescribed for all but patients 17 and 23. Notably, patient 14 was already taken corticosteroids and cotrimoxazole at that time, for still misidentified ENT disease, as GPA had not yet been diagnosed.

Nine patients also received local treatment. Of the 2 patients who were not prescribed medical treatment, patient 20 underwent laser therapy for SGS and patient 9 was not treated because BS was asymptomatic and systemic GPA in remission at that time. The only initial local BS treatment was mechanical dilation, done for patients 1, 4, and 6. Seven patients received local treatment of their SGS: laser therapy for 5, mechanical granulation tissue removal for 2, and/or dilation or local corticosteroid injection for 1 each.

Six (60%) of the 10 patients with BS(s) and 9/16 (56%) SGS patients were prescribed only medical treatments.

#### Systemic Treatments for Relapses

Medications were prescribed for 39/51 (76%) relapses (6/9 [67%] BS and 33/42 [79%] SGS relapses). The most frequently used were corticosteroids, IV or oral cyclophosphamide, azathioprine, and/or subcutaneous or oral methotrexate, which were, respectively, prescribed for 49%, 18% (IV [8%] as often as oral [10%]), 16%, and 14% of the relapses. IV immunoglobulins, infliximab, mycophenolate mofetil, cotrimoxazole, and rituximab were other systemic therapeutic options.

#### Local Treatments for Relapses

Local treatments were prescribed for 35/51 (69%) relapses (7/9 [78%] BSs and 28/42 [67%] SGSs relapses). Rigid bronchoscope dilation was done for 18/51 (35%) relapses (3/9 [33%] BSs and 15/42 [36%] SGSs), a stent was inserted for 7/51 (14%), and laser and/or local corticosteroid injection were used for 6/51 (12%) relapses. Stenting (silicone endoprosthesis) was only prescribed for BSs, and only SGSs were injected with corticosteroids. Laser was used for both TBS types (2/9 [22%] BS and 4/42 [10%] SGS relapses). Resection–anastomosis surgery, mechanical granulation tissue removal, or mitomycin C injection was done, respectively, only in 5, 3, or 2 SGS patients. Patient 16 underwent concomitant dilation and local corticosteroid injection twice. Nine other patients benefited from other local treatment combinations (dilation + stenting, laser + corticosteroid injection, etc.) so that combined local therapy was used for 11 (31%) of the 35 relapses treated locally.

Finally, patients were prescribed concomitant local and medical treatments for 25/51 (49%) relapses.

### Outcomes

Table 4 reports the efficacies of medical and local treatments, alone or combined, to achieve SGS or BS remission, or delay the next relapse. Remission rates after initial treatment or relapse management were 32% for all patients, 53% for BS, and 26% for SGS patients. Their respective mean intervals until the next relapse were 16, 8, and 18 months. Remission was achieved in 46% events treated only medically, without concomitant local procedure(s) (75% in case of BS, 35% in case of SGS). Combined therapies prevented further relapses in 24% cases, local treatment alone in 25%, and 0% in BS patients.

**TABLE 4:**
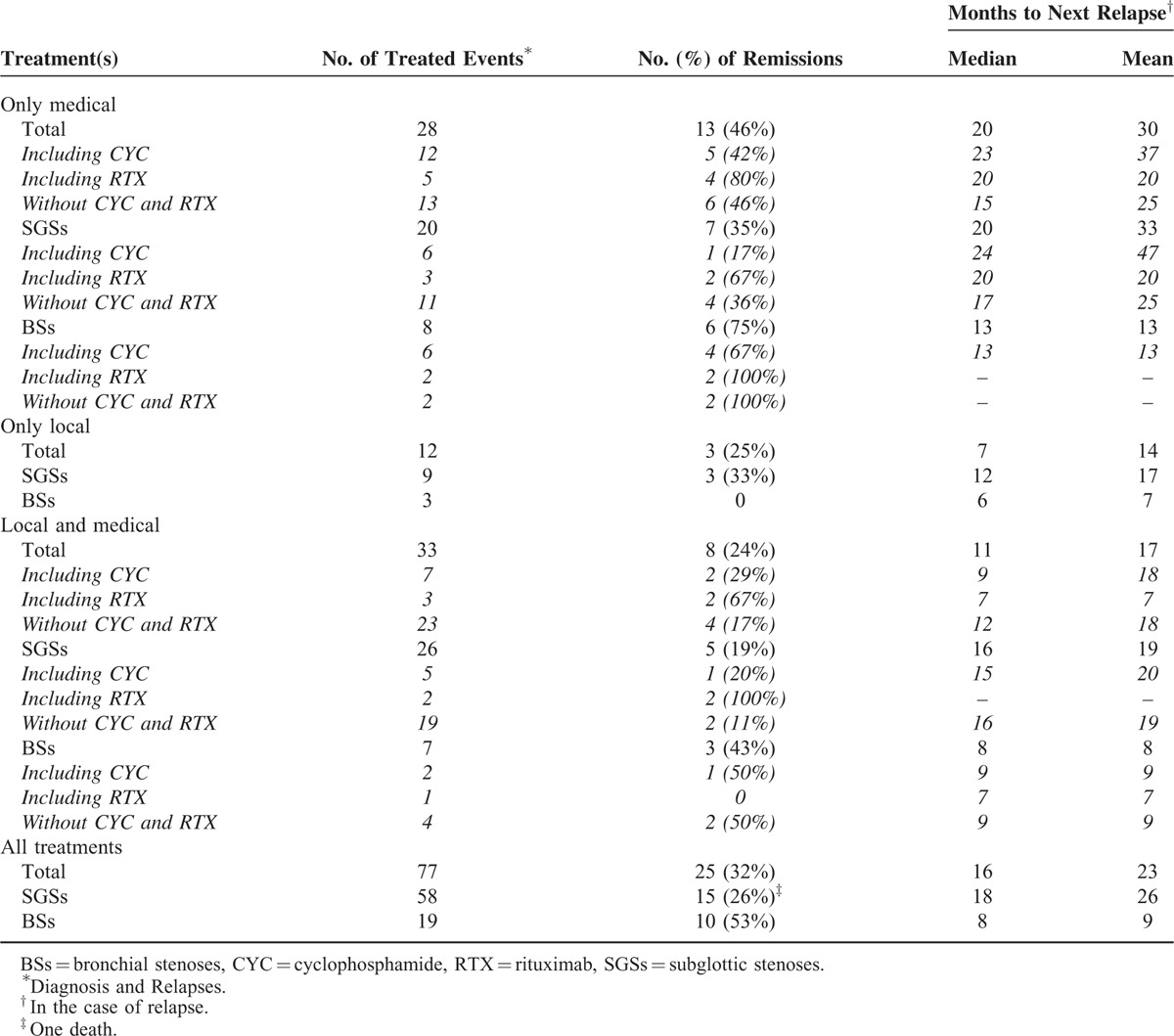
Influence of Treatment Type and Regimen on Attaining Remission or Time to Next Relapse

#### Cyclophosphamide or Rituximab Impact on TBSs

In SGS patients, cyclophosphamide allowed to achieve remission in 17% cases when used without associated local treatment, 20% when part of combined therapy. Seven of all the patients’ 51 relapses occurred under cyclophosphamide (mainly IV, for 5/7 relapses). SGS was diagnosed in patients 13 and 21 while they were receiving IV cyclophosphamide for induction therapy of their GPA. In BS patients, remission was achieved after cyclophosphamide treatment in 50% cases when associated with local procedure, 67% when not. Among the 10 patients with BSs, patient 1 was the only one receiving IV or oral cyclophosphamide at the time of TBS diagnosis.

The remission rate after rituximab infusion without combined local therapy was 80% (67% in SGS and 100% in BS patients). It was 67% when associated with local treatments. Rituximab achieved SGS remission in patients 11, 13, and 18, respectively, after their first, fourth, and eighth relapses, and for patient 15, remission was delayed, appearing only after the second rituximab infusion, given for her sixth relapse. For patient 1, rituximab was unable to prevent an early BS flare, but his SGS then remained asymptomatic for months.

#### Effects of Concomitant Dilation and Local Corticosteroid Injection, Laser Therapy, or Resection–Anastomosis Surgery on SGSs

For patient 16, no subsequent relapse occurred after 2 successive dilation procedures combined with local corticosteroid injection. SGS-remission rates were 23% after any kind of endoscopic procedure (alone or combined with medical treatment), 37% after laser therapy, and 40% following surgical intervention, with respective means of 17 and 19 months before the next relapse for the latter 2 procedures.

#### Complications

Eleven (42%) of the 26 patients developed ≥1 treatment or TBS complication(s). Acute respiratory distress was the most frequent, occurring in SGS patients 11, 12, 15, 18, 19, and 22, and patient 1 with BSs, and was the only complication recorded for SGS patients. In contrast, patients 1, 4, 5, and 9 with BS(s) developed bronchopulmonary infections. In patient 3, whose BS had been stented, the endoprosthesis migrated into the mediastinum. Tracheotomy was never performed. During follow-up, only patient 22 died (acute respiratory failure).

## DISCUSSION

Few papers have focused on TBS in GPA.^2–6,9,12–37^ SGS is the most frequent stenosis type described, but the rare publications on SGS have mostly been case reports.^2–4,6,12–14,16,18–21,24–25,27–33,35,37^ Nonetheless, SGS frequency in larger series was estimated at 16% to 23% of GPA patients.^4,18^ BSs are even less studied and little is known about their epidemiology, clinical features, and therapeutic responses.^2,5,15,17,22,23,26,31,34,36^

Evaluation of our sample confirmed that TBSs, especially SGSs, are unusual GPA manifestations, which do not follow the same evolution as other organ-system manifestations of this vasculitis.

First, our population seems to differ demographically from the general GPA population described in literature. Indeed, the majority of our 26 patients were female, whereas the sex ratio for GPA is known to be 1:1.^38^ Furthermore, they were mostly young, while GPA usually affects patients in their fifth decade. These characteristics were especially true for SGS patients, who were even more frequently female and younger than patients with BS(s), who were demographically closer to the general GPA population. Intriguingly, females also predominated in pediatric GPA patients (male/female ratio of 1:4), with a relative SGS risk of 5 in this population.^39^ Our patients’ systemic manifestations also differed from those usually seen in GPA.^1^ Indeed, they had less frequent renal involvement, regardless of whether they had SGS or BS(s). On the contrary, ENT disease was frequent but pulmonary manifestations (other than BS(s)) were less common in SGS patients. Moreover, our SGS patients were less frequently ANCA-positive and more often MPO-positive than patients with BS(s), who, again, more closely resembled the general GPA population. Finally, tracheal and bronchial histological findings were never GPA-specific, with few granulomatous lesions and little vasculitis.

Our findings agree with those previously reported, that is, the majority of cases were women and frequently young, especially those with SGS. For example, the largest cohort, described in 1998 by Langford et al,^4^ was composed of nearly twice as many women as men (27 vs 16) and the median age at diagnosis was 26 years. More recently, in 2008, Solans-Laque et al^32^ described 6 GPA patients with SGS, 5 of whom were women, whose average age was 33 years. Finally, in Schokkenbroek et al's^9^ retrospective study of the same year, the percentage of females and the mean age at GPA diagnosis, respectively, of the 9 patients investigated were significantly higher (*P* < 0.01) and lower (*P* < 0.05) than those of patients diagnosed with GPA at that institution during the same period. Fewer publications and no large series are available on GPA patients with BS(s). Similarly, our small population may not be representative of all GPA patients with BS(s).

Notably, BSs have been described much less frequently than SGSs. Among our patients, those with BSs were a minority, with 2 patients also having tracheal stenoses. A possible explanation could be that BSs are frequently asymptomatic and discovered fortuitously, during bronchoscopy performed for pulmonary parenchymatous lesions, for example, unlike SGS which rapidly becomes symptomatic (voice changes, noisy breathing, dyspnea, etc.). Thus, BSs could actually be underdiagnosed among GPA patients.

TBS patients’ clinical and immunological GPA characteristics are not well known. Schokkenbroek et al^9^ did not find any differences in other organ involvements between their 9 TBS patients and general GPA population. On the contrary, in accordance with our observations, Langford et al^4^ found renal and pulmonary manifestations to be less frequent in SGS patients. Among the 6 patients described by Solans-Laque et al,^32^ none had renal involvement but ENT disease was usually associated. Concerning ANCA titers, almost all patients reported were ANCA-positive, mostly with anti-PR3 c-ANCA (35/37 [95%]) in Langford et al's cohort.^4^ The somewhat weaker ANCA frequency of SGS patients in our series is surprising.

Otherwise, the absence of TBS-histology specificity was previously described.^2,4,12^ However, according to our findings and those of Hervier et al,^5^ granuloma may be seen more often than vasculitis in bronchial biopsies, but its frequency remains to be confirmed in larger series.

All these observations may lead to considering GPA with TBSs, especially SGSs, particular forms of the vasculitis. Thus, our findings, like those of others, highlight the differences of BSs and SGSs, compared with other organ involvements, in terms of evolution and therapeutic responses.

Our results confirmed that TBSs evolve independently of all other GPA manifestations. Indeed, in the case of secondary TBS diagnosis, GPA was otherwise in remission in half the patients. Above all, a large majority of TBS relapses occurred without any other evolutive GPA organ-system involvement. And, in 81% of the remaining patients with simultaneous GPA and TBS relapses, ENT disease was the only active GPA manifestation. Similarly, ANCA remained negative during half of the TBS relapses and all 3 BS relapses, when they had been sought. These findings are similar to those of other authors over the years,^2,4,5,18^ including for ANCA titers.^2^ For Schokkenbroek et al,^9^ 95% of SGSs appeared when the disease was the less active.

Moreover, TBS relapses were common and recurrent, suggesting the absence of response to classical GPA treatment. Indeed, when diagnosed secondary to GPA, TBSs mostly occurred under GPA therapy. Moreover, for our 26 patients, a large majority had recurrent TBSs, which were often multiple, whereas GPA remained in remission otherwise in 63% of these patients, as previously described.^2,18,19^ However, SGSs and BSs have not yet been compared.

Although the mean number of relapses was the same for our SGS and BS patients, SGSs may be at higher risk of relapse than BSs. Indeed, among our 10 patients with BS(s), only 3 relapsed and 2/3 relapsed only once or twice. The mean number of relapses for this group was biased because of the third, patient 1, who had 6 relapses. Pertinently, he had a particular disease phenotype, with both BSs and SGS, and was thus perhaps closer to patients with only SGS, especially because the latter also relapsed 6 times. This unusual phenotype has been much less described in the literature than the association of BS(s) and dTS(s). Concerning isolated BS(s), which are much more common, relapses are possible but usually few in number. For example, only 2 of the 7 BS patients reported by Hervier et al^5^ relapsed, and only once for both. On the contrary, Tiernan et al^26^ described a 19-year-old woman whose BS relapsed >10 times in 7 years. To compare this mixed-TBS phenotype to patients with only SGS in a large cohort would be of interest to determine whether they exhibit the same demographic, clinical, immunological, and histological features or therapeutic responses.

Assessing the benefit of the different treatment options for SGS and BS management in our study was difficult because of the numerous therapeutic strategies (many systemic and local treatments) which were frequently combined, leading to multiple different associations. The small size of our sample precluded relevant statistical analyses. However, exclusively medical treatments seemed to be more effective, at achieving remission and/or delaying the next relapse, than exclusively local or combined local and medical therapies, especially for BSs. These surprising findings that contradict those of previous large studies should be interpreted with caution. Indeed, the TBSs managed with local or, a fortiori, local and systemic treatment were probably more severe lesions, having more dismal vital and functional prognoses, than those treated only medically, for whom achieving rapid remission was less urgent.

Interestingly, herein, neither laser therapy nor resection–anastomosis surgery was attributed an increased risk of SGS relapse, as was sometimes advanced in the literature.^3–6^ Indeed, it had never been subjected to statistical analyses until now, and some authors even recommended those treatments.^16,18,19,26^ However, prudence is necessary because of the small size of our population and the frequently combined local and medical treatments that, again, do not allow definitive conclusions to be drawn about the safety of laser and surgery to manage SGS.

Unfortunately, dilation combined with local corticosteroid injection, which is highly recommended in the literature,^4,6,21,29,32^ was prescribed for only 1 patient, so we cannot conclude about its contribution to TBS, especially SGS, management. Nonetheless, it was quite effective in our patient 16 who has been in SGS remission since the second procedure, after a long series of relapses. Also, in our cohort, only 7 local corticosteroid injections, without concomitant dilation, were given.

Even though our sample is too small, and the different treatment types too numerous and frequently combined to enable subgroup analyses, we would like to temper the dogma that TBSs do not respond to conventional GPA therapeutic agents. Indeed, cyclophosphamide, which seemed useless against SGSs, may be effective against BSs, again making it more similar to other classical GPA manifestations. Furthermore, rituximab may be of interest for SGS management, as it achieved remission after multiple relapses in our 4 SGS patients for whom it was prescribed. Two different teams had, to date, extolled the virtues of rituximab as treatment for SGS, but each in a single GPA patient.^37,40^

Therefore, whereas endoscopic treatments of TBSs are being supported more-and-more often in the literature, particularly dilation combined with local corticosteroid injection for SGS, they were not associated with better clinical outcomes herein, compared to a more classical medical strategy. Indeed, although cyclophosphamide seems to be poorly effective at treating SGSs, it remains useful for BS management, and rituximab may prove to be an attractive option for SGSs. Of course, these therapeutic assumptions should be viewed with caution, in light of the above-mentioned lack of statistical analyses. Large, prospective, randomized studies are necessary to determine the best management strategy for TBSs, but difficult to achieve because of the rarity of GPA and, a fortiori, of these manifestations.

Concerning SGS follow-up, as previously proposed, flow-volume-loop pulmonary function assessment was shown to be an easy and sensitive technique.^27,29^ PET scan was not considered useful (but was performed on only 2 patients) and MRI was never used. However, the latter was recently shown to be a more informative tool for the management of SGSs, both for morphological follow-up and noninvasive assessment of the status of inflammatory activity.^41^

## CONCLUSION

TBSs are not-so-uncommon GPA manifestations of which physicians should be aware given their marked functional and life-threatening risks. Our observations seem to confirm their specificities of evolving independently of other GPA manifestations and circulating ANCA titers, which may be especially true for SGSs that do not respond to most immunosuppressive regimens and for which local endoscopic management could be particularly contributive, especially for life-threatening disease. Moreover, SGS patients seem to differ markedly from the usual GPA population, with a higher percentage of females, younger age at GPA onset and less frequent PR3-ANCA-positivity. These differences might be explained by a particular pathophysiological process responsible for SGS, whose study may help us better understand and treat this severe GPA manifestation. Concerning rituximab use for TBS management, more and larger prospective studies are needed to determine whether it could be a valid option for the treatment of this challenging condition.
